# New insights into mitochondrial segregation from the Doubly Uniparental Inheritance system in bivalves

**DOI:** 10.1186/s12915-025-02459-6

**Published:** 2025-11-26

**Authors:** M. Iannello, G. Piccinini, F. Salatiello, G. Forni, F. Nicolini, U. Valdrè, M. Martini, J. Martelossi, F. Ghiselli, E. D’Aniello, L. Milani

**Affiliations:** 1 Department of Biological, Geological, and Environmental Sciences, University of Bologna, Bologna, Italy; 2 Department of Biology and Evolution of Marine Organisms, Stazione Zoologica Anton Dohrn, Napoli, Italy; 3 Department of Life Sciences, University of Trieste, Trieste, Italy; 4 Division of Genetics and Genome Biology, School of Biological and Biomedical Sciences, University of Leicester, Leicester, UK; 5 Senckenberg Research Institute and Natural History Museum, Frankfurt, Germany

**Keywords:** Doubly Uniparental Inheritance (DUI), *Mytilus galloprovincialis*, RALA gene, CDK1 gene, Mitochondrial fission

## Abstract

**Background:**

While nuclear genome segregation is well characterized, mechanisms underlying mitochondrial partitioning remain partially obscure, even though its failure can cause developmental arrest or harmful mutations. This knowledge gap invokes the need for new, more suitable model systems to study such mechanisms. Doubly Uniparental Inheritance (DUI) of mitochondria in bivalves is a useful system for such studies. In DUI, sperm mitochondria in male embryos are actively transported across cell divisions to precursors of the germline, and this male-specific pattern depends on maternal factors stored in eggs. The presence of distinct mitochondrial segregation patterns in male and female embryos offers a unique opportunity to investigate the molecular bases of this process.

**Results:**

Here, we leveraged this system by (1) performing RNA-Seq on eggs producing male-biased *versus* female-biased progenies in the Mediterranean mussel *Mytilus galloprovincialis* to identify factors involved in differential mitochondrial segregation; and (2) inferring signatures of convergent evolutionary rate across DUI bivalve genomes to separate segregation-specific factors from those involved in sex determination. We show that differentially transcribed genes across eggs that give rise to either male- or female-biased progeny are predominantly associated with mitochondrial dynamics, cytoskeletal organization, and vesicular trafficking. We also identified multiple long noncoding RNAs—many derived from transposable elements—that might have roles in the regulation of other maternally supplied factors that shepherd paternal mitochondria.

**Conclusions:**

By overlaying clues from expression and sequence evolution, we delineate a conserved protein–protein interaction network of factors that mediate mitochondrial segregation. This study reveals general principles of organelle selection in animals and unveils the contribution of new factors.

**Supplementary Information:**

The online version contains supplementary material available at 10.1186/s12915-025-02459-6.

## Background

The proper segregation and distribution of genetic and cytoplasmic material in daughter cells is crucial during cell division [11]. While the mechanisms underlying nuclear genome segregation are well understood, the processes regulating mitochondrial inheritance remain less clear [61]. Mitochondria are dynamic organelles essential for ATP production via oxidative phosphorylation (OXPHOS), calcium homeostasis, and apoptosis regulation [13]. They carry their own genome, which encodes essential components of the OXPHOS system. Ensuring a proper segregation of mitochondria during cell division is therefore vital to maintain cellular energy production and signalling. This is particularly important during early embryo development, where correct segregation allows an even distribution of mitochondria and their mitochondrial DNA (mtDNA) variants across blastomeres. Such distribution buffers the presence of dysfunctional organelles or pathogenic mtDNA variants by keeping them below the critical threshold required to impair cellular function (the so-called threshold effect; [72]). Conversely, defects in mitochondrial segregation can amplify stochastic genetic drift, leading to an unbalanced representation of the original mitochondrial population [36]. As a result, some blastomeres may inherit too few or dysfunctional mitochondria, potentially leading to developmental arrest [61]. Likewise, imbalanced distribution of mtDNA variants can allow the accumulation and transmission of pathogenic mutations, which may result in severe clinical outcomes [78].

Although the molecular mechanisms underlying mitochondrial transport and segregation are not fully elucidated, it has been hypothesized that they involve the fission/fusion dynamics, and a coordinated remodelling of the cytoskeleton (primarily actin filaments and microtubules; [61, 80]). In the study of mitochondrial dynamics, yeasts have served as powerful models. In *Saccharomyces cerevisiae*, which undergoes asymmetric division, mitochondria are actively transported into the daughter cell along actin cables, revealing key components involved in the process, such as actin, motor proteins, and fission regulators [45]. In mammals, both stochastic and active mechanisms of mitochondrial segregation have been described. During early embryogenesis, mitochondrial fragmentation and dispersion are thought to promote passive, equal partitioning [89]. In contrast, asymmetric divisions in mammalian stem cells have revealed a biased distribution of aged mitochondria, suggesting some sort of quality-control mechanism that preferentially selects mitochondria with optimal metabolic activity in the daughter cell that maintains pluripotency [40].

**Fig. 1:**
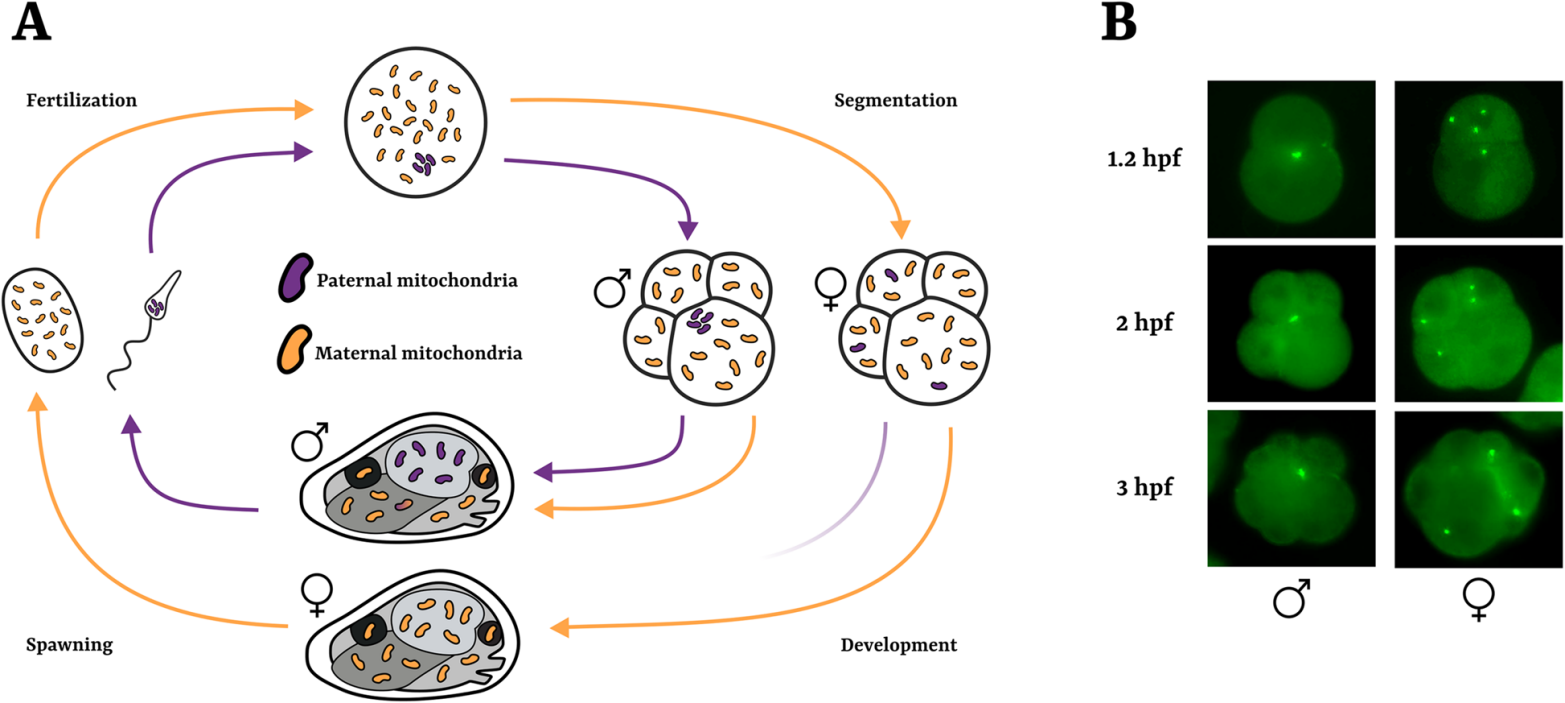
Doubly Uniparental Inheritance (DUI) of mitochondria in bivalves.** A** Schematic cycle of maternal (orange) and paternal (purple) mitochondrial inheritance through generations. In DUI species, two separated mitochondrial lineages are inherited through gametes of different sexes. In female embryos, paternal mitochondria are dispersed, while in male embryos, they remain aggregated and localize close to the cleavage furrow in the D cell (4-cell stage). This aggregated pattern will continue throughout embryo cell divisions and it will be inherited by primordial germ cells: male adults will be homoplasmic for paternal mitochondria in gametes. **B** Fluorescence microscopy pictures of different early embryo stages with paternal mitochondria marked with MitoTracker Green prior to fertilization. In male embryos, the aggregate pattern close to the cleavage furrow is evident; in female embryos, paternal mitochondria are randomly scattered across blastomeres. Hpf = hours post fertilization

In animals more broadly, it remains unclear to what extent mitochondria are actively transported during cell division. This uncertainty may reflect a lack of suitable model to study this process rather than a true absence of such mechanisms [61]. In this context, some bivalve mollusks provide a promising model system for studying mitochondrial segregation. These species display a peculiar mechanism of mitochondrial inheritance called Doubly Uniparental Inheritance (DUI; [27, 28, 95]). Unlike the classic Strictly Maternal Inheritance (SMI) observed in most eukaryotes [75], DUI entails the transmission of paternal mitochondria to the male germline, while maternal mitochondria are typically inherited by both sexes (Fig. 1).

Sperm mitochondria in DUI species exhibit distinct patterns of localization and segregation starting from the first embryo division in at least two major lineages of DUI bivalves (i.e., in Mytilidae and Veneridae; [10, 17, 57, 66]), suggesting that the same process likely occurs as well in other bivalve lineages with DUI. In female-developing embryos, the 4 or 5 mitochondria of the spermatozoon show a dispersed distribution across blastomeres, in contrast, in male-developing embryos, paternal mitochondria aggregate and localize close to the cleavage furrow segregating into the D-blastomere lineage that originates the germline (Fig. 1; [10, 17, 26]). Although DUI is likely specific to bivalves, extensive evidence suggests that it evolved as a modification of ancestral SMI pathways [27, 28]. The coexistence within a single species during embryo development of two mitochondrial segregation modes that can be easily identified through microscopy (as explained below), and the presence of active transport of paternal mitochondria, offer a unique experimental opportunity to identify molecular factors involved in mitochondrial segregation and transport. Indeed, by leveraging this model, it becomes possible to test factors differentially involved in the typical maternal mitochondrial inheritance, observed in DUI females, and in the alternative inheritance observed in DUI males. Such comparative approaches can reveal not only candidate factors specifically involved in DUI, but also more general regulators of mitochondrial inheritance.

The mechanisms underlying such paternal mitochondria transmission are still unclear and no specific factors have been confidently identified at present. This is true both for the mechanisms that ensure homoplasmy for the paternal mitochondrial type in spermatozoa, and for mechanisms underlying the segregation of paternal mitochondria to specific blastomeres during embryonic divisions. A previous work in *Mytilus galloprovincialis* proposed that paternal mitochondria avoid degradation during the overall mitochondrial “purge” of spermatogenesis thanks to a conserved mtDNA sequence that bind an unknown spermatocyte-specific nuclear factor [48]. Despite multiple models have been proposed over time to explain the mitochondrial segregation during the embryonic stage [25, 95, 96], involving several possible candidate factors (e.g., ubiquitin tagging, mitochondrial ORFans; [21, 26, 58, 59, 63], no strong evidence has been gathered yet.

One thing for which researchers appear confident is that the factors determining sperm mitochondrial dynamics in early embryos of DUI bivalves are maternal factors stored in eggs [25, 42, 43, 73, 95, 96]. This is supported by two observations: first, sex-specific mitochondrial segregation begins in the very first embryo divisions (at 2-cell stage), before zygotic genome activation. Second, the sex and mitochondrial fate of the progeny appear to be controlled by the mother [42, 43, 73]. In DUI species, there are females producing up to 100% eggs that will develop females (female-biased eggs) and females producing up to 100% eggs that will develop males (male-biased eggs), independently of the male used for fertilization [42, 43, 73]. While a causal link between mitochondrial inheritance and sex determination has been proposed [6], studies with hybrid crossing and triploid embryos in the DUI species *Mytilus galloprovincialis* suggest that the two processes can be decoupled [43], so they are not causally connected, at least not directly.

In this study, we leveraged the unique mitochondrial inheritance system of DUI bivalves to identify candidate genes involved in mitochondrial segregation. Specifically, we compared transcription profiles between male- and female-biased eggs of the DUI species *M. galloprovincialis* to identify differentially deposited transcripts that may regulate paternal mitochondrial dynamics during early development. By comparing male embryos (which actively retain and traffic paternal mitochondria) with female embryos (where this mechanism is not taking place) provides a unique opportunity to elucidate the molecular factors and mechanisms controlling mitochondrial segregation and transport. Considering that, in addition to mitochondrial segregation, the two biological conditions investigated also exhibit opposite sex-ratio distortion, we sought to distinguish transcripts associated with DUI-specific mitochondrial patterns from those involved in sex determination. Hypothesizing that genes associated with the DUI mechanism may have been subjected to different selective pressures in DUI vs SMI species, we investigated whether differentially expressed genes showed convergent patterns of molecular evolution in DUI species, or whether they interacted with genes that have undergone different evolution in DUI species. By integrating differential expression and evolutionary analyses, we identified a network of interacting proteins potentially involved in mitochondrial transport—highlighting key targets in the mechanisms underlying mitochondrial segregation in animals.

## Results

### Protein Coding Genes (PCGs) involved in microtubule-based movement and lncRNAs show differential transcription in sex-biased eggs

**Fig. 2:**
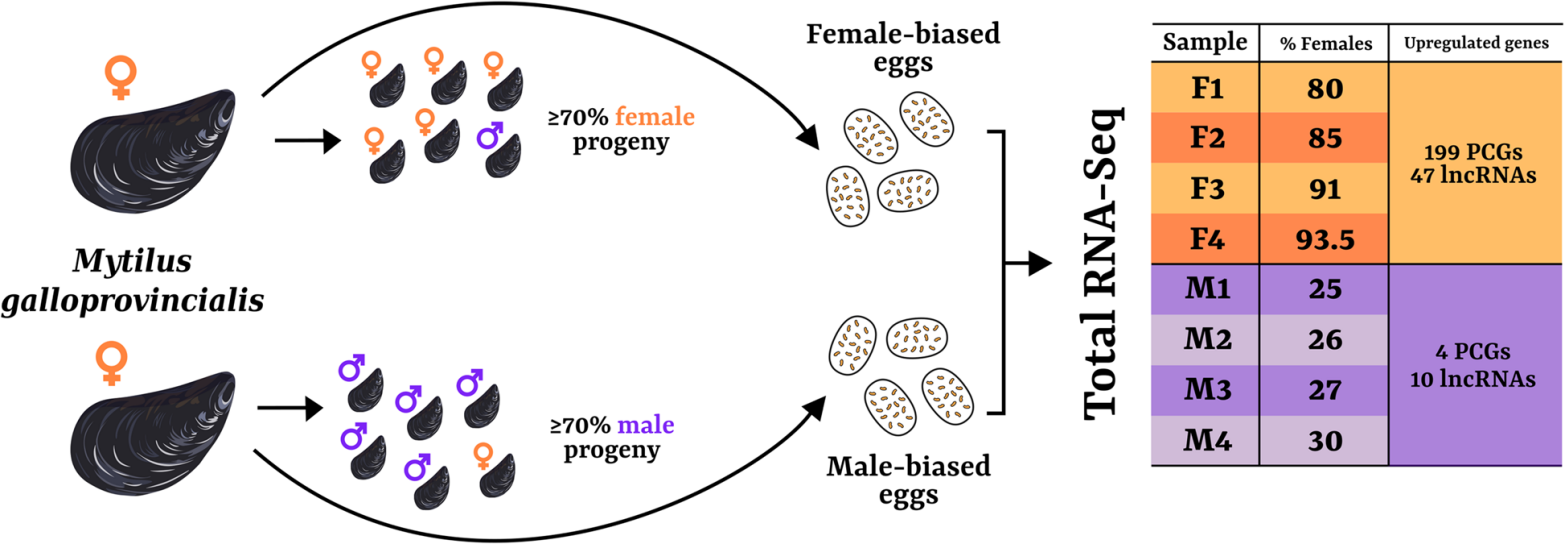
Design and results of the rRNA-depleted transcriptomic analysis. Different females of *Mytilus galloprovincialis* were tested for production of offspring with severe sex-ratio distortions by counting the number of early embryos with aggregated or dispersed patterns of paternal mitochondria (tagged with MitoTracker Green prior to fertilization; see Fig. 1). We then sampled eggs of females producing female-biased (≥ 70% female-developing embryos) and male-biased (≥ 70% male-developing embryos) offspring, and extracted and sequenced their rRNA-depleted transcriptomes (see table in figure). Differential expression analysis with 4 replicates per sex bias revealed 199 PCGs and 47 lncRNAs upregulated in female-biased samples (orange in table), and 4 PCGs and 10 lncRNAs in male-biased samples (purple in table)

To identify potential candidate genes involved in specific mitochondrial segregation in DUI species, we first identified females producing female- and male-biased eggs by inspecting the segregation of paternal mitochondria in oocytes fertilized with spermatozoa previously treated with MitoTracker. Subsequently, the four most extreme male-biased and four most extreme female-biased unfertilized egg samples were sampled and subjected to RNA isolation, rRNA depletion, and RNA-Seq (Fig. 2; offspring sex ratios of all processed samples, quantification of RNA extracted, numbers of raw reads, trimmed reads, and mapping reads are available in Additional File 1: Tables 1, 2, and 3). After removing features with low counts, we ended up with a total of 15,826 features (Additional File 1: Table 4), which have been used to perform DE analyses (see “Differential Expression” in Methods). We found a total of 260 Differentially Expressed (DE) features (Fig. 2 and Additional File 1:Table 5) across male-biased and female-biased eggs (203 Protein Coding Genes, or PCGs, and 57 long non-coding RNAs, or lncRNAs), of which 93 showed also a significant correlation with the sample offspring sex ratio, meaning that their transcription level was directly proportional to the number of males in the progeny (see “Differential Expression” in Methods; Additional File 1:Table 5). The vast majority of DE genes were more transcribed in female-biased samples (199 PCGs and 47 lncRNAs), while a total of 14 features were upregulated in male-biased samples (4 PCGs and 10 lncRNAs; Fig. 2). Among the enriched Biological Process Gene Ontology terms (GO terms; [83]) in these PCGs, we found that microtubule-based movement was by far the most significant (7.5 × 10^−15^; all enrichments for female-biased DE genes in Additional File 1: Table 6). The 4 PCGs showing male-biased expression (MGAL_10B077591, MGAL_10B004966, MGAL_10B004323, and MGAL_10B003489) were annotated respectively as Cyclin-dependent Kinase 1 (CDK1), Manganese-dependent ADP-ribose/CDP-alcohol Diphosphatase, and Ras-related Protein Ral-a-like (RALA), while the latter was uncharacterized (for the complete annotation of DE genes see Additional File 1:Table 7). Moreover, their expression values (as trimmed means of M values, or TMMs, see Methods) were significantly correlated with the sample offspring sex ratio (except for CDK1).

We then tested the presence of Protein–Protein Interactions (PPIs) among these PCGs, and we observed that the protein products of the DE genes show more interactions among each other than what expected by chance (PPI enrichment *p*-value = 1.11 × 10^−4^, number of edges = 52, number of expected edges = 29). A total of 55 proteins interacted with each other, constituting 13 small networks of interactions, with a median node degree of 2 (Additional File 1:Table 8).

LncRNAs, on the other hand, were identified through genome annotation but they had no associated functions. Some of the DE lncRNAs had significant hits against the MirGeneDB 3.0 database of metazoan miRNAs [15], suggesting a potential role as precursor-miRNAs (specifically, 4 out of 10 male-biased and 7 out of 57 female-biased lncRNAs; Additional File 1:Table 9A). Since lncRNAs are known to potentially act as pre- or post-transcriptional regulators also in sequence-specific manners [85, 94], we also back-BLASTed them against the *M. galloprovincialis* genome to look for potential pairing sites, focusing only on strong hits included within other genes (see “Differential Expression” chapter in Methods). Indeed, 15 lncRNAs had multiple hits within introns of *M. galloprovincialis* PCGs (ranging from 2 to 1823 hits). Moreover, 10 of these lncRNAs had significantly more hits included within the sequences of DE genes with respect to random expectations (Additional File 1:Table 9B). When aligning the top 20 hits of these elements with their flanking sequences we observed that they originate from longer homologous regions spread through the genome (Additional File 2:Fig. 1A). Despite the absence of clear Trasposable Element -related (TE-related) structural features like Target Site Duplications, Terminal Inverted Repeats and coding regions, the consensus sequence are highly similar to *Mytilus* Kolobok TEs (Additional File 2:Fig. 1B) and show a tandem-like structure (Additional File 2:Fig. 1C). These lncRNAs may thus originate from the frequent multimerization and excision events of Kolobok transposons, as previously proposed in *Mytilus coruscus* and other bivalves—events that are characteristic of these elements and may give rise to complex non-autonomous elements [46].

### Convergent constrained evolution underlies multiple genes in DUI species

**Fig. 3:**
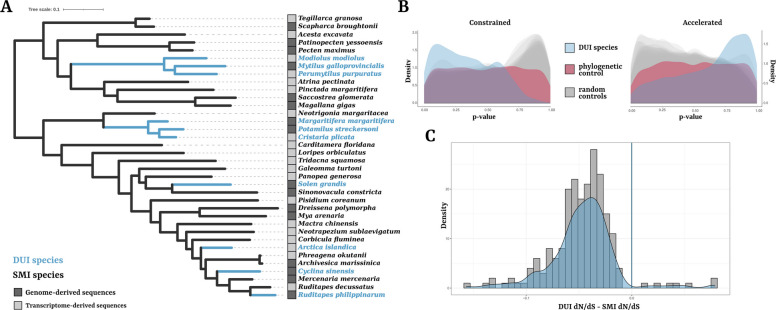
Convergent selection analysis in DUI vs SMI species.** A** Phylogenetic relationships of the 36 bivalves included in the analysis. Branch lengths were optimized on a reference species tree obtained from the literature (see Additional File 1:Table 10) using an alignment of 967 OGs containing at least 32 species. Light blue branches refer to DUI species. **B** *p*-value distributions of the TRACCER replicates, divided for OGs considered convergently “constrained” or “accelerated” by the software. In blue is the *p*-value distribution by tagging DUI species; in red is the *p*-value distribution of the phylogenetic control; in gray are the *p*-value distributions of random controls. There is a clear enrichment for lower *p*-values only in OGs with convergently “constrained” sequence evolution by tagging DUI species. **C** Distribution of ΔdN/dS between DUI and SMI species for significant OGs in the branch test of Codeml. For nearly all OGs, the values are lower than 0 (blue vertical line), meaning a stronger purifying selection (lower dN/dS) in the branches belonging to DUI species with respect to those belonging to SMI ones

To investigate whether DE PCGs may have a role in the differential segregation of mitochondria in DUI species, we analyzed their molecular evolution in comparison to SMI species. To do this, we used 36 online-available bivalve proteomes, comprising 6 separate occurrences of DUI in the bivalve tree (see “Genomic dataset of DUI and SMI species” chapter in Methods; Additional File 1: Table 10). We analyzed a total of 8972 single-copy orthologues comprising at least 15 species and at least 4 DUI species (Fig. 3A, Additional File 1:Tables 10 and 11; see “Ortholog identification” chapter in Methods for details). To test the presence of convergent evolution in DUI species, we compared amino acid evolutionary rates with the TRACCER software [84] and found 354 genes showing convergent “constrained” evolution in DUI species, and 89 with convergent “accelerated” evolution (Fig. 3B; Additional File 1: Table 12; see “Evolutionary Analyses” chapter in Methods). We found an enrichment for significant *p*-values in genes showing convergent “constrained” evolution in DUI species, compared to both phylogenetic and random controls (Fig. 3B; see “Evolutionary Analyses” chapter in Methods). Differently, we found less genes with convergent “accelerated” evolution in DUI species compared to results obtained with randomly selected species (Fig. 3B). Since we could not exclude that significance was driven only by random effects in “accelerated” genes, we removed them from the subsequent analyses, while we further investigated those with convergently “conserved” protein evolution.

TRACCER uses amino acid sequence; therefore, it is not a direct test for the selective pressures acting on the genes. To test that in the OGs identified as “constrained” by TRACCER, we used CODEML (from PAML suite; [92]) that allow to estimate the ratio between nonsynonymous and synonymous substitution rates (dN/dS) for a codon alignment as a proxy of the selective pressure acting on that gene. With the branch-model of CODEML (see “Evolutionary Analyses” in Methods), we found that 206 of these also showed a convergent stronger purifying selection in DUI species, and we will refer to them as Convergently Evolving genes, or CE genes (Fig. 3C; Additional File 1:Table 13). These same genes were enriched for signals of coevolution with the mtDNA (significant Evolutionary Rate Covariations with mitochondrial PCGs; chi-square *p*-value < 1 × 10^−5^; Additional File 1:Tables 14 and 15; see “Evolutionary Rate Covariations with mitochondrial genes” chapter in Methods), suggesting many of them might be associated to mitochondrial biology. Among the enriched GO terms, we found Biological Processes involved in centrosome cycle, spindle assembly, and Cdc42 protein signal transduction and Cellular Components associated with HAUS complex and mitotic spindle microtubule (all enrichments in Additional File 1:Table 16). When testing for the presence of PPIs across CE genes, we did not find significant results (PPI enrichment *p*-value = 0.06, number of edges = 31, number of expected edges = 23; Additional File 1:Table 17).

### CE genes have a significant number of interactions with DE genes

For 79 DE genes, we could find homologs in the OrthoGroups of the Convergent Evolution analysis (others were discarded during the OG filtering or were not included in any OG at all). Across these, only 3 showed convergent selection in DUI species (namely Mitotic-spindle Organizing Protein 2, Coiled-coil Domain-containing Protein 171, and Structural Maintenance of Chromosomes Protein 2), and this number was not higher than expected by chance. However, when we investigated the presence of PPIs in the combined set of DE and CE genes, we found that the number of interactions was much higher than what expected by change (PPI enrichment *p*-value = 6.46 × 10^−5^, number of edges = 146, number of expected edges = 104). The enrichment for interactions was even higher than that obtained for the two sets of genes separately. Overall, a total number of 131 proteins (products of 54 CE and 77 DE genes) interacted forming two large networks (composed respectively of 59 and 30 proteins) and 11 smaller networks (less than 8 proteins each; Additional File 1:Tables 18 and 19).

**Fig. 4:**
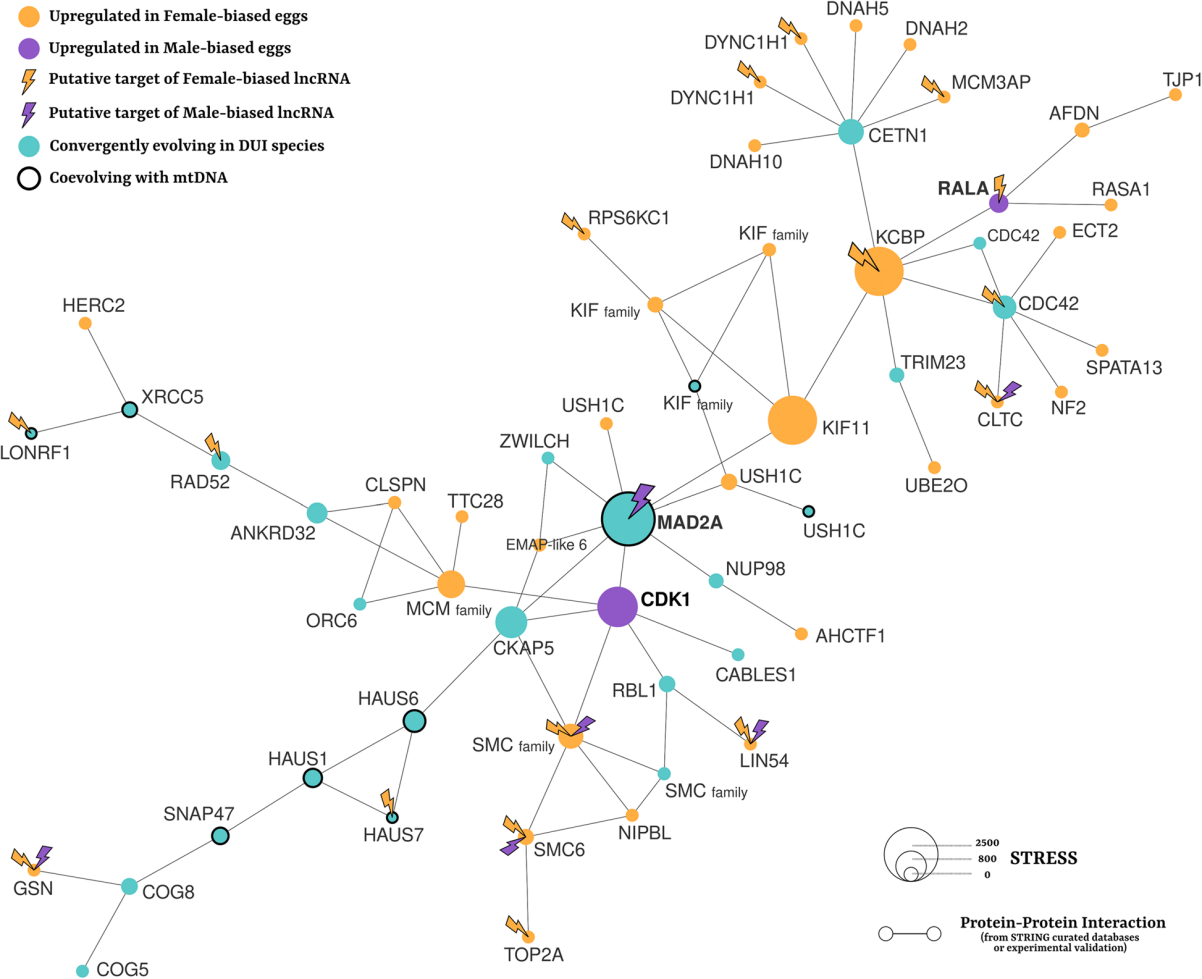
Largest subnetwork of Protein–Protein Interactions (PPIs) between the products of Differentially Expressed (DE) genes in sex-biased eggs and Convergently Evolving (CE) genes in DUI species. The number of PPIs (estimated by STRING considering only “curated databases” and “experimental validation”) between these two sets of gene products is significantly higher than expected (*p*-value = 6.46 × 10.^−5^). The figure depicts the subnetwork with the highest number of PPIs: nodes represent proteins, edges represent PPIs; in orange are products of genes upregulated in eggs that will develop mostly embryos with dispersed paternal mitochondria; in purple those upregulated in eggs that will develop mostly embryos with aggregated paternal mitochondria; in light blue are products of genes with stronger purifying selection in DUI species with respect to SMI species; node sizes are proportional to Stress values; nodes with thick black outline are products of genes whose branch length significantly correlate with those of mitochondrial genes, as a proxy of potential coevolution with the mitochondrial genome. CE nodes have significantly higher values of Degree (number of connected edges) and Stress (a measure of connectivity importance)

We focused our analyses on the largest network (Fig. 4; Additional File 1:Table 20), which showed the highest number of PPIs and including products of male-biased PCGs (namely CDK1 and RALA). Of the 10 DE lncRNAs that have significantly more hits within DE genes with respect to random expectations, 7 (2 male-biased; 5 female-biased) had also significantly more hits within DE genes included in this very network, and 2 of them (upregulated in female-biased eggs) contained anti-sense hits within the RALA gene. By looking at the functional enrichments (Additional File 1:Table 21; Additional File 2: Fig. 2), we found that this network was enriched in Biological Processes such as “microtubule-based movement”, “organelle organization”, “Cdc42 protein signal transduction”, “protein localization”, “cell cycle”, “spindle assembly”, and “chromosome organization”. Moreover, products of CE genes had statistically higher Degree and Stress, suggesting that such proteins have a higher number of connections and are crucial to connect different submodules of the network (Additional File 1:Table 22). Differently, DE gene products show a higher neighborhood connectivity, implying that they are characterized by more local connections.

## Discussion

The co-occurrence in the Bivalvia class of two systems of mitochondrial inheritance (i.e., the DUI and SMI system) represents a fertile testing ground for biological queries such as animal mitochondrial inheritance and the coevolution between mitochondrial and nuclear genomes [27, 28, 33, 67, 77]. In the present study, we focused on the mitochondrial inheritance mechanism, leveraging the early development of *M. galloprovincialis*. In this DUI species, it has been observed how, in a mother-dependent way, offspring can show extreme sex-ratio distortions, with instances of all-female or all-male progenies [42, 43, 73]. Moreover, in *Mytilus*, the sex of the offspring can be experimentally established at the first embryo division by observing the distribution pattern of sperm mitochondria; indeed, in male embryos, the mitochondria of spermatozoon midpiece localize near to the cleavage furrow within the D-cell lineage, whereas in female embryos they are randomly dispersed among blastomeres (Fig. 1; [10, 17]).

Here, we isolated eggs from mothers that predominantly produced embryos with opposite mitochondrial distribution pattern, and sequenced their rRNA-depleted transcriptomes (Fig. 2). We identified 203 Protein Coding Genes (PCGs) and 57 lncRNAs that were differentially transcribed between the two conditions, most of which were upregulated in female-biased eggs (only 4 PCGs and 10 lncRNAs were upregulated in male-biased eggs). The finding of Differentially Expressed (DE) lncRNAs with transposable element-like (TE-like) signatures and multiple hits/copies within the genome, including a higher than-expected number of DE genes, provide a hint for regulatory mechanisms that have not been previously investigated in bivalves, neither in the study of sex determination nor of mitochondrial inheritance. Regulatory mechanisms based on lncRNAs are various, complex, and not yet thoroughly characterized, involving both sequence-specific and protein-mediated binding, as well as transcriptional, splicing, and translational interactions [7, 38, 85]. However, TEs can be intimately associated with lncRNA evolution. For example, through (i) the transcriptional alteration of existing lncRNAs, (ii) the transcriptional activation of novel non-coding regions [38], or even by (iii) evolving themselves in ncRNAs, with also roles in developmental regulation (e.g., [31]). Considering that an association of lncRNAs to the regulation of microtubule assembly has been proposed (e.g., [52]), we think that this overlooked regulatory aspect in bivalve development may have a role in mitochondrial segregation and is worth deeper investigation.

Among the maternal transcripts of oocytes, we identified factors that may be involved both in sex determination and in the differential segregation of paternal mitochondria. Previous studies in *Mytilus* involving triploids and hybrid crosses assessed how maleness and presence of paternal mitochondrial genome can be decoupled, so they are not causally linked [43]. Given our focus in mitochondrial inheritance mechanisms only, we performed analyses of molecular evolutionary convergence across 6 independent occurrences of DUI in bivalves to narrow down the subset of genes that are more likely involved in this process (Fig. 3). Indeed, since the sex determination mechanisms are shared with SMI species while paternal mitochondria transmission is not, we expect the convergent evolutionary signals in DUI species to mostly concern genes involved in the modification of mitochondrial inheritance rather than in sex determination. We identified 206 genes that displayed a convergent stronger purifying selection in DUI species with respect to SMI species and that are also enriched for signals of coevolution with mtDNA and microtubule-based cytoplasmic movements.

When we tested if DE genes in sex-biased eggs overlapped with Convergently Evolving (CE) genes, we found that almost none of them did. However, genes convergently selected in DUI species might not necessarily be differentially transcribed in different sexes in the early embryos, but might rather represent key members of pathways involved in mitochondrial dynamics whose balance depends on the up- or downregulation of other factors. Following this rationale, we annotated the number of Protein–Protein Interactions (PPIs) between protein products of the two sets of genes: we found that it was statistically higher than expected (*p*-value = 6.46 × 10^−5^), and even higher than considering the number of PPIs in the two sets separately. This means that the products of DE genes and CE genes in DUI species have a significant degree of interaction. Moreover, in the networks we obtained, products of genes convergently selected in DUI species were averagely characterized by higher degree and stress, suggesting them being key components of network connectivity. This finding highlights the contribution of integrating molecular evolution and differential transcription analyses to achieve a multi-level picture of the process investigated.

In the largest subnetwork (Fig. 4), we identified the products of several genes previously associated with mitochondrial dynamics in eukaryotes. Among these, we found different motor proteins, such as 5 subunits of Dynein Heavy Chain and 5 Kinesin-like proteins, and proteins associated to microtubule-based movements, which are known to have a key role in the positioning and partitioning of mitochondria in animals and yeasts [61, 80]. For instance, we identified multiple subunits of the HAUS/Augmin complex (as genes with DUI-specific selective pressures and signals of coevolution with mitochondrial genome), that was initially considered as a regulator of mitotic spindle dynamics in animals [29, 50], but whose involvement was also associated to mitosis-free microtubule organization: e.g., in plant cortical microtubule network [55] or in axonal polarity, that is crucial also for neuronal mitochondrial distribution [74, 87].

Most of the DE genes in our PPI network share a higher expression in female-biased eggs, suggesting that in male embryos the mitochondrial aggregate segregation pattern might be achieved mostly by the negative regulation of the microtubule localization machinery and the upregulation of few factors. In the main PPI network we identified (Fig. 4), two proteins showed transcriptional upregulation in male-biased eggs, and they represent good candidates worth investigating: CDK1 and RALA. The transcription level of the latter is also significantly correlated with the sex-ratio distortion of the different samples, meaning that its transcription is directly proportional to the percentage of male-producing eggs. Additionally, the introns of the *rala* gene in *M. galloprovincialis* include anti-sense copies of two TE-like lncRNAs that are upregulated in female-biased eggs and whose expressions are correlated with the sample sex ratio (MGAL_10NCA010049 and MGAL_10NCA061360; Additional File 1:Table 9B). The presence of opposite transcriptional regulation between a gene and its anti-sense lncRNAs in the two conditions we analyzed provides hints that lncRNAs may have an active role in the regulation of mitochondrial segregation. Although this is just speculative at the moment, the role of lncRNAs in such a topic may deserve deeper investigations.

Among their multiple functions, homologs of RALA and CDK1 are known to be involved in mitochondrial network regulation during mitosis, specifically by contributing to selective localization (by RALA) and activating phosphorylation (by CDK1) of the Dynamin-related Protein 1 (DRP1), a core regulator of mitochondrial fission [39, 81]. Interestingly, in mammals, regulation mediated by Cyclin-dependent Kinases has also been associated to axonal anterograde movement (toward the plus end of the microtubule, i.e., toward the synapsis, [71]), and a direct role of DRP1 in the axonal anterograde movement, specifically of mitochondria, has also been assessed [4]. Moreover, in primates, RALA was observed to be selectively localized at the cleavage furrow during cytokinesis, where the plus ends of microtubules are located [12, 14]. The upregulation of CDK1 and RALA in male-biased eggs, together with the downregulation of dynein subunits which participate in transport toward the minus ends of microtubules [93], strongly suggests potential molecular candidates involved in the selective localization of paternal mitochondrial aggregate at the cleavage furrow of male D-lineage blastomeres.

As far as we know, these are the strongest candidates that have ever been proposed for the molecular machinery responsible for the peculiar mitochondrial inheritance system of *M. galloprovincialis*. These results provide new candidate genes that need to be tested also at the protein level with immunofluorescence analyses on the embryos, but also on other DUI species. Indeed, in the present study the PPI network of these genes was retrieved also thanks to molecular evolutionary convergence throughout 6 separate occurrences of DUI in the Bivalvia class, providing it with a significance wider than species-specific results.

The identification of many genes in the network with functions in mitochondrial dynamics, transport, and segregation, highlights how assessing the involvement of such pathways and such genes in the mechanisms of mitochondrial segregation in DUI species can provide indications about the mechanism of mitochondrial segregation in animals. The molecular machinery of mitochondrial segregation in dividing cells is yet to be characterized thoroughly, and how and to what extent mitochondria are selected. Here, we found hints for the localization and movement of mitochondria across multiple cell divisions through DRP1-associated mechanisms. A causal link between mitochondrial fragmentation and mitochondrial segregation in the germline was already highlighted in other animal species, such as fruit flies and mice, and mitochondrial fragmentation is considered to be of primary importance for symmetric mitochondrial partitioning and for mitochondrial variants segregation, [24, 64]. Interestingly, it has been shown that DRP1 has a main role in controlling the mitochondrial redistribution and partitioning during embryonic cleavage in mouse, and deletion of DRP1 in mouse zygotes causes marked mitochondrial aggregation and biased mitochondrial inheritance [24]. The fact that two activators of DRP1 are central in our network structure and are differentially expressed in eggs that will produce mostly embryos with the aggregate pattern and a different mitochondrial segregation in the germline, suggests a role of genes underlying mitochondrial fragmentation in early embryo mitochondrial segregation, and brings out CDK1 and RALA as key factors regulating the process.

Our network also provides some additional candidate genes that show a key role in the network composition in terms of Stress and Degree (Fig. 4). Among these, we highlight the Mitotic Spindle Assembly Checkpoint Protein MAD2A, a component of the spindle-assembly checkpoint that prevents the onset of anaphase until all chromosomes are properly aligned [49]. This gene has a key role in our network, having the highest Stress and Degree, its non-coding regions include anti-sense copies of a TE-like lncRNA upregulated in male-biased eggs (MGAL_10NCA069327), it is subjected to convergent stronger purifying selection in DUI species, and it has a significant evolutionary rate correlation with mtDNA, which may suggest that this gene has a role in mitochondrial organization and segregation (Fig. 4). Altogether, this tempts us to question if it might have a role in preventing cells from entering the anaphase until a proper mitochondrial segregation is ensured as well. It is known that an improper partitioning of mitochondrial content in daughter cells may result in severe consequences [78], suggesting that an organelle distribution checkpoint is theoretically plausible as much as a chromosome partitioning checkpoint. Even though, as far as we know, there is no direct evidence of a link between MAD2A and mitochondrial segregation, it has been observed that DRP1 knockout in mice oocytes leads to organelle aggregation and meiotic arrest [24]. Such evidence highlights how cell division and proper mitochondrial partitioning are intimately linked and corroborates the hypothesis that MAD2A may have a role in preventing the onset of cell division until not only a proper chromosome segregation is achieved, but also a correct partitioning of mitochondria. This is just speculative at the moment, but it would be interesting to functionally test such a hypothesis in future.

## Conclusions

The findings of this study support the idea that the investigation of the DUI system may provide new perspectives for the study of mitochondrial segregation in animals. In our opinion, the integration of molecular evolution analyses, differential transcription, and protein–protein interaction networks has been a crucial part of this work, and it allowed us to achieve a more comprehensive and multi-level view of the investigated process. Overall, we believe that the identified interactions across cytoskeleton-associated proteins, regulation of cell cycle process, and regulation of mitochondrial fragmentation reflect, at least partially, the complexity of the mechanisms underlying mitochondrial segregation in dividing cells, and offer new promising candidates for genes involved in such a process.

Specifically, we proposed the mitochondrial fission-related proteins RALA and CDK1 as key elements involved in the selective positioning of paternal mitochondria in the cleavage furrow of male embryos of *M. galloprovincialis*. Moreover, we identify multiple lncRNAs with differential distribution in male- and female-biased eggs that might be involved in gene expression regulation, representing additional investigational units that need to be taken into consideration when approaching the DUI system. Lastly, we also propose the further investigation of the role that the protein MAD2A might have in promoting proper mitochondrial distribution in the DUI system, but also more broadly in SMI species.

## Methods

### Sampling of sex-biased eggs

Sexually mature specimens of *M. galloprovincialis* were purchased from IRSVEM SRL (Bacoli, Napoli, Italy). Gamete spawning and fertilization were carried out in March 2023 as previously described in Jimenez-Guri et al. [34] (spawning season of *M. galloprovincialis* occurs approximately from February to April in the Tyrrenian Sea). The segregation of paternal mitochondria was traced by fertilizing eggs with sperm pre-labelled with the vital dye MitoTracker Green FM (M7514, Invitrogen), as previously described in Milani et al. [57]. Embryos were grown at 18 °C at a concentration of 250 eggs per milliliter until the developmental stage of interest. Specifically, the sex ratio was assessed by the distribution of sperm-derived mitochondria visualized through fluorescence microscopy at 2–4–8-cell stage embryos (approximately 2.5–3 h post fertilization) with a Zeiss Apotome Microscope (Carl Zeiss AG, Oberkochen, Germany).

Three different operators observed the patterns of mitochondria distribution for each of 21 fertilizations, for a mean number of 29 pictures analyzed and 182 embryos counted per sample. Each operator calculated the ratio of embryos showing aggregated vs dispersed patterns of paternal mitochondria (i.e., male-developing vs female-developing embryos, respectively). The median of ratios obtained from the operators was used as a proxy of the sex bias in the offspring of each sample. Samples showing ≥ 70% of aggregated paternal mitochondria were considered as “male-biased”, those showing ≥ 70% of dispersed mitochondria were considered as “female-biased” (see Additional File 2: Figs. 3A and 3B for an example of male-biased and female-biased offspring, respectively). Between 12,500 and 25,000 unfertilized eggs for each female with sex-biased progeny were collected and kept in TRIzol™ at − 80 °C until RNA extraction (Additional File 1: Table 1).

### Library preparation and sequencing

Total RNA extraction of the most skewed 4 male-biased and 4 female-biased egg samples was performed using the TRIzol™ protocol. The RNA Clean & Concentrator™−5 (Zymo Research) was used to clean the extracted total RNA. Cleaned RNA was quantified with a Qubit fluorometer using the Qubit RNA BR kit. Libraries of rRNA-depleted transcripts were prepared using the Zymo-Seq RiboFree Total RNA Library Kit (Zymo Research), with the following setups: input RNA 230 ng, 15 min of depletion reaction (Section. 2.1 of the protocol, step 4), 12 PCR cycles (Section 4.1). After library preparation, dsDNA was quantified with Qubit using the Qubit dsDNA HS kit and with a Bioanalyzer using the Agilent DNA 12000 LADDER kit. Samples were then shipped to Zymo Research Corporation (Irvine, CA, USA) and sequenced using Illumina NovaSeq 6000.

### Differential expression

Quality of the raw reads was visualized using FastQC [2] and raw reads were trimmed using TRIMMOMATIC v0.39 [5], with the following parameters: *LEADING:3 TRAILING:3 SLIDINGWINDOW:25:33 MINLEN:75*. Paired trimmed reads were mapped against the available annotated *M. galloprovincialis* genome (GCA_900618805.1) using HISAT2 [44]. Mapped reads were then filtered using SAMtools [51] with the following parameters: *-f 0* × *2 -F 256 -q 30*. FeatureCounts [53] was used to count mapped reads for each feature in our samples. For each sample, counts were filtered with the *filtered.data* function of NOISeq package [82] on R v4.3.1 [70] to exclude transcripts showing less than 2 counts per millions (cpm) in all samples. Raw counts were then normalized using the trimmed mean of M values (TMM). Differential expression (DE) between female- and male-biased samples was calculated using the *noiseqbio* function of NOISeq. Genes were considered as differentially transcribed if the q value was > 0.95.

Besides showing a qualitative difference (male- or female-biased offspring), samples also had a quantitative variation, related to the sex ratio of the offspring. This means that, in each sample defined as sex-biased, a certain percentage of eggs that would produce the opposite sex was present (up to nearly 30% for the less skewed conditions), together with sex-specific transcripts. For this reason, for each DE transcript, we tested for correlations between the distribution of TMMs and sex ratios in all 8 samples. Significantly correla 2ted results were considered as more probably associated to sex-specific differences.

Protein Coding Genes (PCGs) were identified based on genomic annotation; their domains and Gene Ontology (GO) terms were annotated with InterProScan v5.45.80 [37]. Long non-coding RNAs (lncRNAs) were identified through genome annotation, but they had no associated functions. To see whether some could be precursor miRNAs, we BLASTed [9] them against MirGeneDB 3.0, an online database of metazoan miRNA genes. To see whether lncRNAs could be involved in pre- or post-transcriptional regulation in sequence-specific manners [85, 94], we back-BLASTed them against the *M. galloprovincialis* genome to look for potential pairing sites: we considered hits longer than 100 bp, covering more than 50% of the lncRNA, and with an identity higher than 90%. To narrow down potential regulatory roles, we considered only hits included within genes (or 100 bp up/downstream). When more than five hits were identified in the genome for a given lncRNA, we manually screened the alignments of the top twenty hits, along with 1000 bp of flanking regions, to assess their potential relationship with transposable elements (TEs), building up a consensus sequences with the advance consensus maker online tool (https://hfv.lanl.gov/content/sequence/CONSENSUS/AdvCon.html) and looking for characteristic structural features such as target site duplications and terminal inverted repeats, following the protocol described by Peona et al. [68]. Additionally, the elements were screened for homology with previously described TEs deposited in RepBase [3] with Censor online.

### Genomic dataset of DUI and SMI species

To test the presence of genes with convergent molecular evolution in DUI species compared to SMI species, we analyzed PCGs from a genomic and transcriptomic dataset of bivalves. Species were chosen in order to maximize the phylogenetic distribution of bivalves, and to ensure the highest number of DUI occurrences across the different taxonomic groups (preferring genomes over transcriptomes when available). A total of 15 annotated genome assemblies were downloaded from various public resources (Additional File 1: Table 10). When possible, at least two genomes from two different genera were selected for a specific order to encompass the taxonomic diversity of bivalves; for each genus, the best genome according to its BUSCO score (on the metazoan_odb10 dataset; [56]) and assembly statistics was chosen. Isoforms were removed from each genome annotation file using the dedicated perl script from the AGAT toolkit v0.7.0 [19].

To encompass the limited availability of genomes for certain bivalve clades (such as Carditidae, Lucinidae, and Pinnidae), we included in our dataset proteomes retrieved from transcriptomic data. We used transcriptomes assembled by Piccinini et al. [67] for 9 species, and we additionally assembled de novo 12 species (Additional File 1: Table 10). For the latter, raw reads were downloaded from NCBI SRA and trimmed with Trimmomatic v0.39 with the following parameters: *LEADING:3 TRAILING:3 SLIDINGWINDOW:25:33 MINLEN:75*. Paired-end trimmed reads have been assembled using Trinity v2.6.6 [30] with default parameters. CD-HIT [32] with default parameters was used to remove transcript redundancy. Following Piccinini et al. [67], we removed contaminants from the newly obtained transcriptomes using a DIAMOND search [8] against the NCBI non-redundant protein database (nr), with the “*staxids*” flag. E-fetch (NCBI E-utilities package) was used to extract the full taxonomic lineage for each DIAMOND hit, and only transcripts showing the first best hit against a lophotrochozoan were kept. Open Reading Frames were retrieved from transcriptomes using TransDecoder v5.5.0 (https://github.com/TransDecoder/TransDecoder/), with minimum amino acid length of 50. The prediction of Opend Reading Frames was implemented with BLASTp and HMMER searches [35] against the UniProt [86] and the Pfam [62] databases, respectively. The completeness of the proteomes was evaluated with BUSCO v5 with the Metazoa ortholog set (as implemented in gVolante: https://gvolante.riken.jp/, [65]).

### Ortholog identification

We ran OrthoFinder v2.3.11 [22] on our proteome dataset, using default settings and the DIAMOND *ultra-sensitive* mode. Multi-copy OrthoGroups (OGs) were split to single-copy OGs using DISCO v1.3.1 [90]. Single-copy OGs were aligned using MAFFT v7.475 (*–maxiterate 1000 –localpair*, [41]). Alignments were then trimmed with BMGE v1.12 [18] to remove regions with high entropy (*BLOSUM30, h* = *0.75*). Moreover, we excluded all alignment positions that were missing in more than 50% of the species, and all sequences that were composed of more than 80% of gaps. Trimmed amino acid sequences were retro-translated to trimmed nucleotide sequences with a custom script. The *quality_check_trees.py* script available in Dixon and Kenkel [20] was used to remove from downstream analyses all OGs whose phylogenetic tree did not respect the monophyly of bivalve subclasses (Pteriomorphia, Palaeoheterodonta, and Heterodonta,gene trees built with FastTree v2.1.11; [69]). In cases when one or two species alone were responsible for the validation fail, we removed such species from the OG, and realigned the OG. We then further selected OGs based on their representativeness of the dataset: we kept for subsequent analyses only OGs with at least 15 species and at least 4 DUI species. Considering that the investigated species are phylogenetically distant, orthologous sequences can be highly divergent. This may affect ortholog clustering, resulting in a dataset biased toward more conserved genes. No solution exists to bypass this problem, and, consequently, only a subset of genes could be analyzed in the present study. For each OG, the longest amino acid sequence was used to perform a functional annotation: we BLASTed them against NCBI nr to annotate gene names, when available, and we used InterProScan v5.45.80 to annotate GO terms and domains.

### Evolutionary analyses

To test the presence of convergent evolution in DUI species, we used TRACCER (Topologically Ranked Analysis of Convergence via Comparative Evolutionary Rates; [84]). TRACCER investigates gene trees to perform Relative Evolutionary Rates across all pairs of species exhibiting two conditions of a given trait (in this case, DUI and SMI species), comparing them with expected values from a reference species tree. Without making assumptions on the ancestral state of the trait, the software uses branch lengths of each gene tree to identify signals of convergent evolution in the specified set of species (in this case, DUI species), discerning between genes with convergent “accelerated” or “constrained” evolution.

For each OG, we calculated amino acid branch lengths on the fixed topology of the species tree (Species tree based on literature: see Additional File 1: Table 10). The best-fitting model for each alignment was inferred with ModelFinderProtein as implemented by IQTREE v2.2.5 [60]. We optimized branch length on the species tree topology with RAxML-NG v1.1.0 (*-blopt nr_safe*, [47]). Optimized gene trees were fed to TRACCER by tagging DUI species to identify convergently evolving genes across them. Branch lengths for the reference species tree needed by TRACCER were inferred on the concatenated alignment of OGs containing at least 32 species (967 OGs,model selection and partitioning with ModelFinderProtein, exploring 25% of all possible partition combinations; branch length inference with RAxML-NG).

To determine if the number of genes showing convergent evolution in DUI species was significantly higher than what expected by chance, we additionally ran 50 replicates of TRACCER tagging 10 randomly selected SMI species (instead of DUI species; random controls). Moreover, to check for any putative phylogenetic bias that might have driven the analyses, we performed an additional control by tagging species closely related to DUI ones (phylogenetic control). The distribution of *p*-values of analyses performed on DUI species and controls were then plotted and compared using R.

TRACCER infers evolutionary convergence based on amino acid branch lengths. To perform a test on selective pressures acting on OGs identified as “constrained” by the software, we used CODEML (from PAML suite; [92]) on their retro-translated trimmed alignments. CODEML calculates the ratio between nonsynonymous and synonymous substitution rates for a codon alignment as a proxy of the selective pressure acting on that gene. Here, we wanted to test for different selective pressures in different species,therefore, we used branch models. For each OG, we compared the likelihood of two models: a null model with a uniform dN/dS for all branches, and a nested model with a different dN/dS value for DUI species clades with respect to the background (here called “DUI branch model”). With Likelihood Ratio Tests, we evaluated the best-fitting model for each OG, and when “DUI branch model” was better, we calculated the difference between the dN/dS of the background and that of DUI species branches (as a proxy of stricter or more relaxed purifying selection acting on DUI species with respect to SMI ones). OGs for which “DUI branch model” was better were considered as convergently evolving (CE) genes in DUI species.

### Evolutionary Rate Covariations with mitochondrial genome

To identify OGs that might be involved in mitochondrial functions, we used the proxy of Evolutionary Rate Covariation (ERC). Briefly, if branch lengths between two gene trees with a fixed topology show a significant correlation, this is a signal of a putative coevolutionary link between them, suggesting some level of functional association [16, 54]. Significant ERCs have been observed for instance between mitochondrial and nuclear subunits of OXPHOS complexes [67, 77, 91]. However, such test can be also used at a genome-wide level to identify functionally linked gene modules that are not necessarily in physical contact but that co-participate in the same biological pathways, and that were therefore subjected to similar evolutionary rates and pressures along the same tree branches [23]. Here, we tested ERCs between each OG tree and the mitochondrial tree to identify genes that are more likely associated with mitochondrial biology.

To infer mitochondrial branch lengths, we used all mitochondrial PCGs. For each species, we used a custom script to extracted mitochondrial PCGs from either assembled transcripts or genomic contigs, using DIAMOND against a reference database of all bivalve mitochondrial genes from NCBI followed by TransDecoder to identify open reading frames. When possible, we prioritized the extraction of PCGs from the transcriptomes and genomes used in this study, to have mitochondrial and nuclear sequences belonging to the same individuals. In most genomes, however, mitochondrial contigs were not present, and in those cases we directly downloaded sequences from the reference mitochondrial genome on NCBI. Branch lengths of the mitochondrial tree were calculated from the concatenated amino acid alignment of mitochondrial PCGs on the fixed species tree topology (phylogenetic methods as described before for OGs).

Using a custom script, species root-to-tip distances of each OG tree were tested for correlation against the root-to-tip distances of the mitochondrial tree. Values were adjusted for phylogenetic biases by calculating their Phylogenetic Independent Contrasts (PICs; *pic* function of *ape* R package), on which we tested the fitting of a linear regression. PICs were calculated using the reference species tree used for TRACCER.

### Integration of signals from convergent evolution and differential expression

To investigate the presence of Protein–Protein Interactions (PPIs) across genes showing signatures of selection in DUI species (CE genes) and genes showing differential transcription in female- and male-biased eggs (DE genes), we used STRING [79], with “databases” and “experiments” as active interaction sources and “medium confidence (0.400)” as minimum interaction score. We chose *Mizuhopecten yessoensis* as a reference species, since it was the only bivalve present in STRING.

We analyzed the largest STRING network using Cytoscape 3.10.2 [76]. We used the “Analyze Network” tool to investigate common network metrics, focusing in particular on ”Degree”—i.e., the number of edges connecting a node to its direct neighbors—and “Stress”—which quantifies how much a node is necessary to maintain the network [88]. Finally, we tested the differential contributions of CE and DE genes to the network composition (see “Functional enrichment and statistical analyses”).

### Functional enrichments and statistical analyses

To test the presence of functional enrichments in our genes of interest, we performed GO term enrichments with the *TopGO* R package (*elim* algorithm; [1]). Enrichments were performed using different backgrounds, depending on the analysis: we used the total set of transcribed genes as background when testing GO enrichments across DE genes,and we used the whole set of investigated OGs when testing GO enrichments in CE genes. All statistical analyses were conducted on R v4.3.1. The correlation of sex biases and transcription level was tested with the *cor.test* function (spearman method), and Fisher’s tests with the *fisher.test* function. Finally, we evaluated the significance of differences in network metrics distributions using the *wilcoxon.test* function of *stats* R package.

## Supplementary Information

Additional file 1: Supplementary Tables 1-20. TabS1 - Percentage of female and male developing embryos from each egg sample. TabS2 – Quantification of rRNA-depleted extraction for each sample. TabS3 – Sequencing and mapping results. TabS4 – Expression quantification for each feature in each sample. TabS5 – Differential expression between female- and male-biased samples. TabS6 – GO term enrichment in DE PCGs. TabS7 – annotation of DE PCGs. TabS8 – Network metrics of STRING-inferred PPIs across DE PCGs. TabS9 – LncRNAs annotation and analyses. TabS10 – Evolutionary analyses Dataset. TabS11– Statistics of OrthoGroup assignments. TabS12 – Results of TRACCER analyses. TabS13 – Results of CODEML analyses. TabS14 – Results of Evolutionary Rate Covariation with mitochondrial genes. TabS15 – Statistical test for overrepresentation of genes covarying with mitochondrial genes among CE PCGs. TabS16 – GO term enrichment in CE PCGs. TabS17– Network metrics of STRING-inferred PPIs across CE PCGs. TabS18 – Network metrics of STRING-inferred PPIs across CE and DE PCGs combined. TabS19 - Cluster association for each genes showing protein-protein interactions in network of DE genes and CE genes. TabS20 - Network metrics of the largest PPI subnetwork. TabS21 – GO term enrichment for components of the subnetworks (DE and CE genes separately). TabS22 - Kolmogorov–Smirnov tests evaluating the differential contributions of DE and CE to network metrics distributions.

Additional file 2: Supplementary Figures 1-3. FigS1 - Example of the origination of a lncRNA (MGAL_10NCA066482) from a putative Kolobok transposable element. FigS2 - Functional annotation of the protein-protein interaction subnetwork discussed in the manuscript. FigS3 - Early stage embryos from differently biased samples.

## Data Availability

Raw reads generated for this work are available in the NCBI Sequence Read Archive (SRA; [https://www.ncbi.nlm.nih.gov/sra] (https:/www.ncbi.nlm.nih.gov/sra) ; BioProject PRJNA1281357). Custom scripts used in this paper are available at the following github repository: [https://github.com/MariangelaIannello/Mitochondrial-segregation/] (https:/github.com/MariangelaIannello/Mitochondrial-segregation).

## Abbreviations

CE: Convergently Evolving
DE: Differentially Expressed
DUI: Doubly Uniparental Inheritance (of mitochondria)
ERC: Evolutionary Rate Covariation
GO: Gene Ontology
lncRNA: Long non-coding RNA
mtDNA: Mitochondrial DNA
OG: OrthoGroup
PCG: Protein-Coding Gene
PIC: Phylogenetic Independent Contrast
PPI: Protein-Protein Interaction
SMI: Strictly Maternal Inheritance (of mitochondria)
TE: Transposable element
TMM: Trimmed Mean of M value
