# Supplementary material for: New insights into mitochondrial segregation from the Doubly Uniparental Inheritance system in bivalves
Source: BMC Biol. 2025 Nov 26;23:371. doi: 10.1186/s12915-025-02459-6 (PMC12750733; doi:10.1186/s12915-025-02459-6)
Supplement: Supplementary file 2 — Additional file 2: Supplementary Figures 1-3. FigS1 - Example of the origination of a lncRNA (MGAL_10NCA066482) from a putative Kolobok transposable element. FigS2 - Functional annotation of the protein-protein interaction subnetwork discussed in the manuscript. FigS3 - Early stage embryos from differently biased samples. [file 12915_2025_2459_MOESM2_ESM.pdf]

Supplementary Figures

A

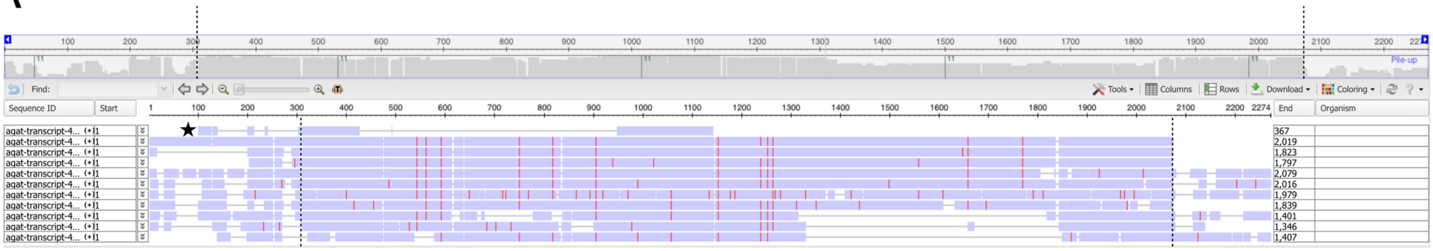

B

Name	From	To	Name	From	To	Class	Dir	Sim	Pos/Mm:Ts	Score
MGAL_10NCA066482#DNA/KolobokP	1	766	KolobokE-4_MyEd	3391	4071	Interspersed_Repeat	d	0.9334	2.3571	1556
MGAL_10NCA066482#DNA/KolobokP	767	1124	KolobokD-11_MyEd	6629	6985	Interspersed_Repeat	d	0.8743	2.3889	772
MGAL_10NCA066482#DNA/KolobokP	1127	1819	KolobokD-11_MyEd	2744	3402	Interspersed_Repeat	d	0.9041	2.0455	1486

C

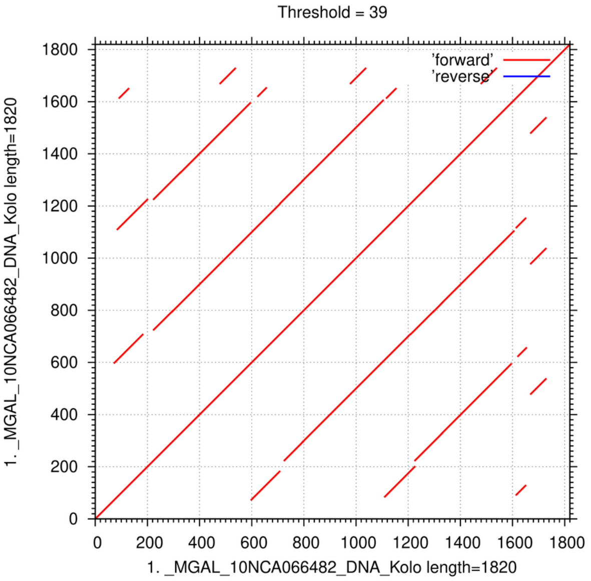

Supplementary Figure 1. Example of the origination of a lncRNA (MGAL_10NCA066482) from a putative Kolobok transposable element. A: Alignment of the lncRNA MGAL_10NCA066482 with 9 other genomic hits and their flanking regions. Dotted lines indicate the boundaries of the element characterized by a drastic decrease in coverage and alignment identity. The star highlights the original transcript. **B:** CENSOR results of the reconstructed consensus sequences. **C:** Self-alignment of the consensus sequence reveals a tandem-like structure.

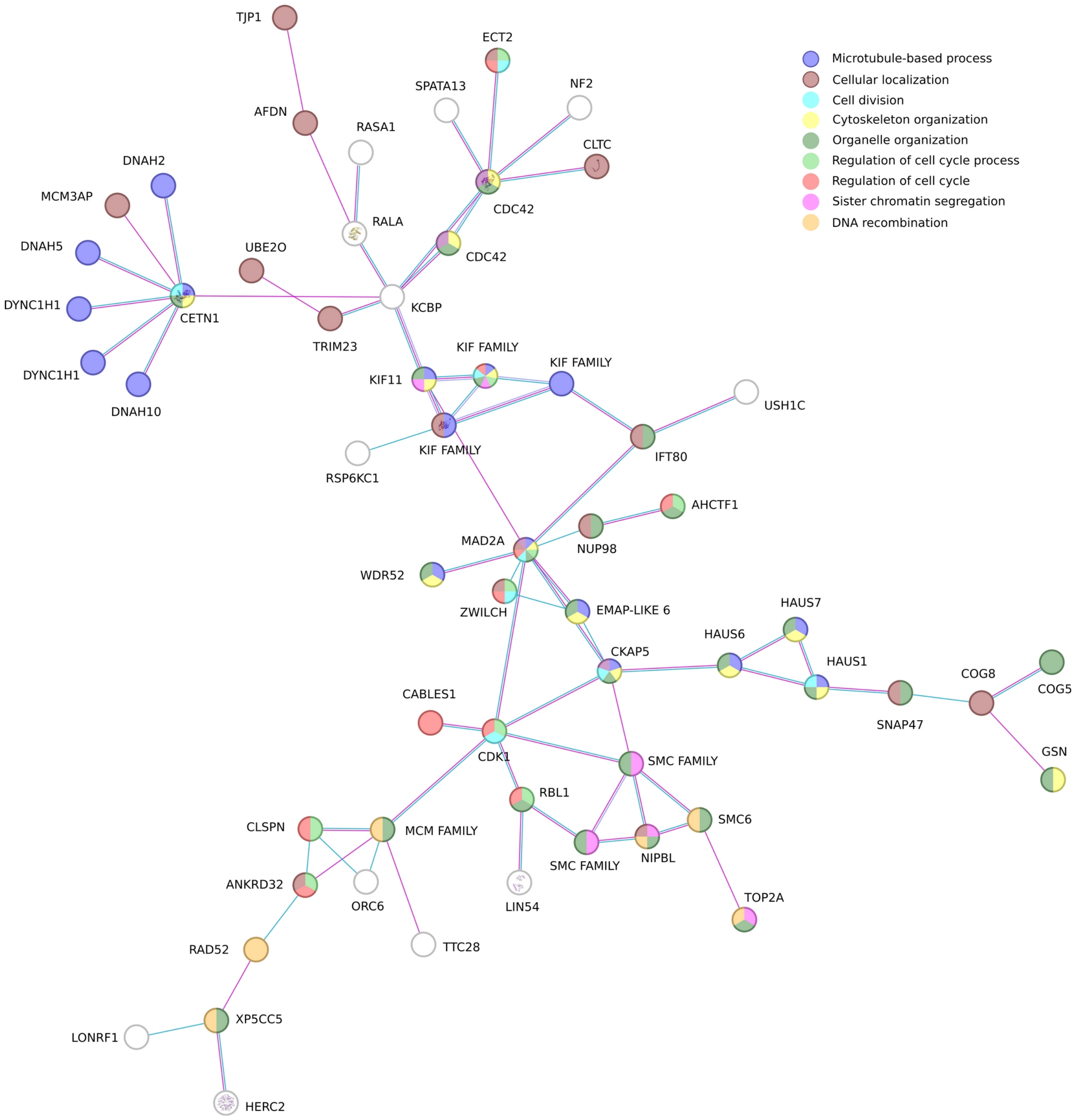

Supplementary Figure 2. Functional annotation of the protein-protein interaction subnetwork discussed in the manuscript. The figure depicts the subnetwork with the highest number of PPIs (same as Figure 4 of the main text): nodes represent proteins, edges represent PPIs. Colors indicate functional annotations retrieved from STRING (see legend).

A B

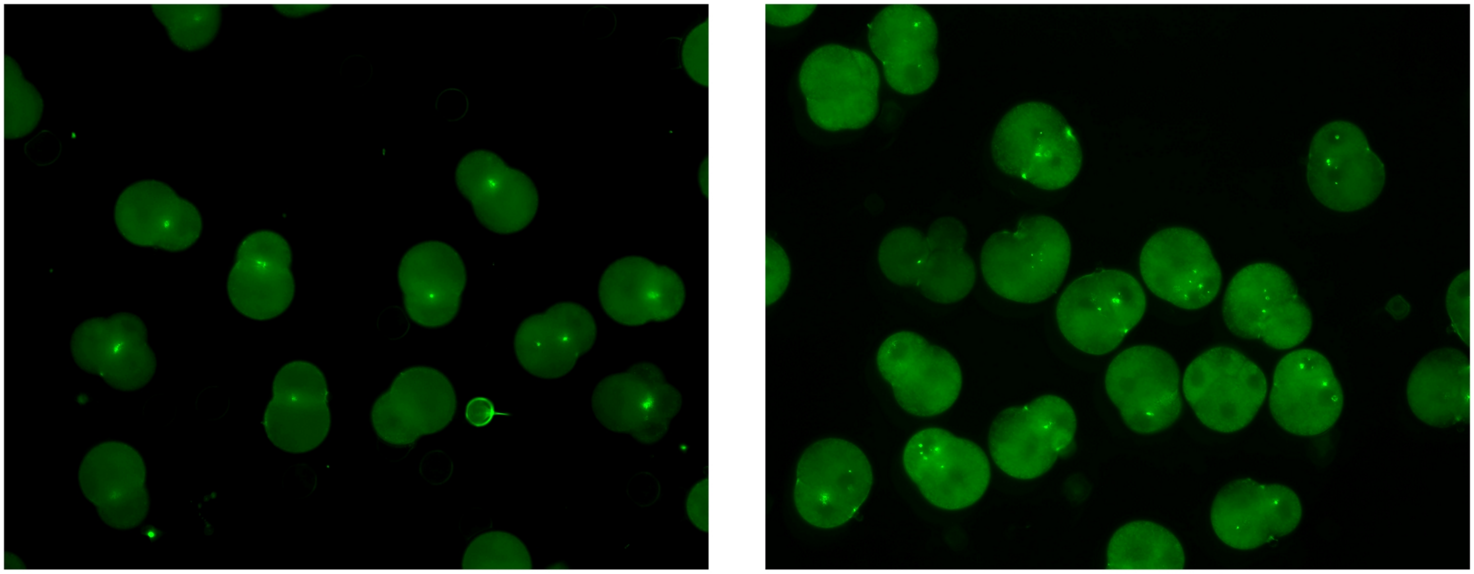

Supplementary Figure 3. Early stage embryos from differently biased samples. Images captured with Zeiss Apotome Microscope (Carl Zeiss AG, Oberkochen, Germany). Sperm were tagged with MitoTracker Green prior to fertilization (green in images). **A:** example of progeny of a male-biased sample; nearly all embryos captured show the aggregate pattern of paternal mitochondria localized at the cleavage furrow of the first cell division. **B:** example of progeny of a female-biased sample; in nearly all embryos paternal mitochondria are randomly scattered through blastomeres.
